# Health-related quality of life and its influencing factors in adult patients with localized scleroderma - a cross-sectional study

**DOI:** 10.1186/s12955-020-01386-0

**Published:** 2020-05-12

**Authors:** Anna Lis-Święty, Alina Skrzypek-Salamon, Irmina Ranosz-Janicka, Ligia Brzezińska-Wcisło

**Affiliations:** Medical University of Silesia, Chair and Department of Dermatology, School of Medicine in Katowice, Francuska 20/24, 40-027 Katowice, Poland

## Abstract

**Background:**

Localized Scleroderma (LoS) is an autoimmune connective tissue disease that affects skin and less commonly subcutaneous tissues. The illness occurs in children and adults, and may have a serious impact on health-related quality of life (HRQoL). The goal of this study was to explore what factors might deteriorate scores on HRQoL measures in adult LoS patients.

**Methods:**

Detailed information on the demographic and clinical features of LoS patients was collected. The HRQoL was assessed using Skindex-29 and Short form 36 (SF-36) questionnaires.

**Results:**

Thirty three women and seven men with LoS were enrolled. Female gender negatively influenced scores for the emotion subscale of Skindex-29. Multiple-factor linear regression analysis confirmed, as with single-factor analysis, that the causes of low SF-36 physical component score (PCS) were subjective symptoms (pruritus, pain, paresthesia), musculoskeletal manifestations and older age at the time of survey, while rural area of residence negatively affected the SF-36 mental component score. Additionally, single-factor analysis showed that the SF-36 PCS was related to the LoS cutaneous assessment tool (LoSCAT) summary score.

**Conclusions:**

Apart from a clinical presentations, sociodemographic characteristics, including older age, female gender and living conditions, may impair HRQoL of LoS patients. Further studies that will examine the role of these factors for physical and mental functioning of adults with LoS are needed.

## Introduction

Localized scleroderma (LoS) is an autoimmune disorder characterised by inflammation and sclerosis of the skin and less commonly subcutaneous fat, in some cases only affecting the fasciae, muscles and bones. It is not a life-threatening illness, but health related quality of life (HRQoL) of patients is often negatively influenced [1]. The early skin lesions are erythematous plaques that become progressively indurated. After several months to years they may resolve but skin and/or deeper tissues atrophy and hyper/hypopigmentation remain. Additionally, linear and generalised subtypes are usually associated with functional impairment i.e. arthralgia and/or arthritis, joint contractures of the affected limb, limb length discrepancy and physical disability [1, 2]. Neurologic symptoms (e.g. seizures/epilepsy, headaches/migraines, stroke) and/or ophthalmology conditions such as uveitis may occur in LoS of the head or face (en coup de sabre subtype, progressive hemifacial atrophy) [3]. Although the linear subtype, musculoskeletal, neurologic and ophthalmologic involvement predominate among children, there are several reports of low health related quality of life (HRQoL) in adults patients [4–11]. Klimas et al. [5] reported that skin-specific HRQoL of adult LoS patients was worse than those with non-melanoma skin cancer, vitiligo, and alopecia. What is more, LoS impacted in the emotions and mental health domains of HRQoL, similarly to rheumatoid arthritis or systemic lupus erythematosus [5]. The clinical variables most influencing HRQoL were pruritus or pain related to LoS lesions and extracutaneous manifestations [4–7, 11]. Nonetheless, there is still known very little about the physical, mental and emotional functioning of patients with LoS. Demographic features and socioeconomic status, that constitute a group of factors strongly affecting the HRQoL [12, 13], were not investigated in most researches on LoS.

The objective of this study was to evaluate the HRQoL and relate it to socio-demographic and clinical aspects of adult onset LoS.

## Methods

### Patients

This cross-sectional study was conducted on patients with LoS representing a range of disease severity (mild to severe), gender (female and male) and age (over 18 years of age). Study participants were recruited from the Dermatology Department at the Medical University of Silesia in Katowice. The patients were diagnosed with LoS according to the classification of Kreuter et al. [14] with a biopsy specimen when necessary. Data were collected by a physician using a structured questionnaire for demographic (age, gender, place of residence, educational level, employment status, marital status) and clinical information (disease subtype, age at the disease onset and at the diagnosis, recurrences, extracutaneous manifestations, subjective symptoms e.g. itch, pain, burning or pricking sensation, coexisting disorders, C-reactive protein level and erythrocyte sedimentation rate). Educational level was measured using 4 possible responses classified as primary, lower secondary, upper secondary and tertiary level, representing 8, 11, 12–13 and minimum of 15 years of formal education, respectively. A physical examination was performed in all patients and all of them were evaluated by consultant ophthalmologist and neurologist and those with arthralgia also by a rheumatologist. Activity of the disease and tissue damage was assessed with LoS cutaneous assessment tool (LoSCAT). This tool is a validated physician-derived measure that consists of two components: the modified LoS skin severity Index (mLoSSI) and the LoS skin damage index (LoSDI) [15–17]. With the use of the mLoSSI disease activity is scored from 0 to 162 based on the evaluation of the intensity of erythema (ER) and skin thickness (ST), appearance of new lesions or enlargement of an existing lesions within 1 month (N/E) in eighteen anatomic sites. Similarly, LoSDI quantifies the tissue damage based on the evaluation of dermal atrophy (DAT), subcutaneous atrophy (SAT) and dyspigmentation (DP) severity. A new LoSCAT composite score (based on 2 above-cited indexes) was used to classify the disease severity. To calculate this firstly introduced global index of skin involvement – LoSCAT summary score, scores of the mLoSSI and LoSDI were added together.

### HRQoL

The HRQoL was evaluated using Skindex-29 (with permission from Janowski Konrad, Ph.D.) and Short-form 36 questionnaires (SF-36, license number QM038248) [18, 19]. Patients were asked to complete both questionnaires regarding past 4 weeks.

The Skindex-29 is a skin-specific, validated instrument that contains 29 questions. Every question is rated on a 5-point Likert scale and scored as 0, 25, 50, 75, or 100 for each available answer choice: never, rarely, sometimes, often, or always. The points are averaged into three subscale scores from 0 to 100: emotions, functioning, and symptoms. Higher numbers indicate a worse score for each subscale. Skindex-29 cutoff scores for mildly, moderately, and severely impaired HRQL (symptoms: 39, 42, 52; emotions: 24, 35, 39; functioning: 21, 32, 37; overall: 25, 32, 44; respectively) are proposed by Prinsen et al. [20, 21].

The Short Form Health Survey (SF-36v2) is a generic tool widely used to assess the level of HRQoL [22]. From this questionnaire, a physical component score (PCS) and a mental component score (MCS) can be calculated. The questionnaire can also be used to measure eight domains to give more specific information about a patient’s HRQOL: physical functioning (PF), role limitations due to physical health problems (RP), bodily pain (BP), general health perceptions (GH), vitality (VT), social functioning (SF), role limitations due to emotional problems (RE), and mental health (MH). The results ranged from 0 to 100, with higher values reflecting better HRQoL. The score of 50 corresponds with the average HRQoL, the scores below 50 reflect lower HRQoL, and the scores above 50—higher up to excellent HRQoL [23].

### Statistical analysis

Descriptive statistics were expressed mainly as medians and interquartile ranges (and also means and standard deviations for clinical data) for continuous variables, and as numbers and percentages for categorical variables. The Shapiro-Wilks test revealed that the distribution of variables was often non-normal, so Spearman’s correlation, Kruskall-Wallis’s and U Mann-Whitney’s tests were used when necessary to explore the relation of each individual variable on the HRQoL. The calculated Spearman’s rank correlation coefficient (r) was evaluated using the Student’s t-test. All variables that showed a relationship (defined as *P* < 0.05) at a univariate level were then included in multivariable linear regression models. To remove “worst” predictors early and to leave only “important” predictors, a backward elimination method was used. Categorical variables such as subjective symptoms and musculoskeletal manifestations were defined as dummy variables, taking the value of 1 if found, and 0 if not. For the place of residence, the rural area of residence was considered the reference category.

All data were analyzed using Statistica 12.0 software (StatSoft, Inc., Tulsa, Oklahoma, USA) and PQStat (v.1.6.2; PQStat, Poznań, Poland) with *p* < .05 being statistically significant.

## Results

Tables 1 and 2 show demographic data, disease – related characteristics and HRQoL of the study subjects. Women comprised 82.5% (33 of 40) of patients included in the study. The average age of the participants was 49.1 ± 18.3 years. Mean age of LoS onset was 42.4 ± 19.4 years. Mean overall LoS duration was 6.6 ± 11.0 years. The most frequently represented subtypes were plaque and atrophoderma of Pasini and Pierini, in 45 and 20%, respectively. The majority of patients had active disease (87.5%) and the first appearance of symptoms (80%). Musculoskeletal manifestations of LoS occurred in three cases: one woman with mixed (coexistence of linear and plaque variants) and the other one with generalized LoS reported arthralgias as the man with linear LoS of the limbs who also had joint contractures. Neurologic or ocular manifestations of the disease were absent in the whole sample. Subjective symptoms (itch, pain, tightness or burning sensations in the affected area) and comorbid medical conditions were present in 5.5 and 62.5% of patients, respectively. The greater number of them lived in urban areas with fewer than 100.000 people (67.5%), had secondary education (60%) and were married or cohabited (77.5%); more than a half (52.5%) were employed.

**Table 1:** Demographic data and disease – related characteristics of 40 patients

Feature	Value
Female sex, n (%)	33 (82.5)
Age at disease onset (years), Median (IQR)	40.5 (29.75–57)
Age at survey (years), Median (IQR)	46.5 (33.75–64.35)
Disease duration (years), Median (IQR)	3.5 (1–10)
Place of residence), n (%)	
Rural	7 (17.5)
Urban, less than 10,000 of people	3 (7.5)
Urban, less than 100,000 of people	24 (60)
Urban, 100,000 or more people	6 (15)
Educational level, n (%)	
Primary	3 (7.5)
Lower secondary	12 (30)
Upper secondary	12 (30)
Tertiary	13 (32.5)
Working (emloyment) status, n (%)	
Employed	21 (52.5)
Unemployed	7 (17.5)
Student	2 (5)
Pensioner	10 (25)
Marital status, n (%)	
Married	30 (75)
Widowed	1 (2.5)
Divorced	2 (5)
Cohabited	1 (2.5)
No relationship	1 (2.5)
Body surface area affected by LoS (%), Median (IQR)	4.25 (1.875–8)
Active disease, n (%)	35 (87.5)
Without remission	26 (65)
Reccurent disease 1st relapse	6 (15)
Reccurent disease 2nd relapse	3 (7.5)
Inactive disease	5 (12.5)
LoSCAT summary score, Median (IQR) Subtypes, n (%)	13.5 (14–25)
Plaque	18 (45)
Atrophoderma of Pasini and Pierini	8 (20)
Generalized	7 (17.5)
Mixed	4 (10)
Linear	3 (7.5)
Musculoskeletal manifestations, n (%)	3 (7.5)
Symptoms (e.g. pruritus)	21 (52.5)
Comorbid medical conditions, n (%)	25 (62.5)
Hypertensio arterialis	9 (22.5)
Diabetes type 2	5 (12.5)
Gastroesophageal reflux	3 (7.5)
Hashimoto disease	2 (5)
Hand eczema	2 (5)
Rheumatoid arthritis	2 (5)
Other	5 (12.5)

*IQR* interquartile range, *LoSCAT* localized scleroderma cutaneous assessment tool

**Table 2:** Health-related quality of life of patients with localized scleroderma

	Mean (SD)	Median (IQR)	Range
Skindex-29			
Symptoms	14.35 (±5.11)	13 (11–18.25)	7–28
Function	19.15 (±8.39)	16 (13–21.75)	12–53
Emotions	23.10 (±8.48)	21 (17–26)	11–50
Short-form 36			
PCS	42.24 (±8.29)	44.70 (37.43–48.34)	23.14–54.16
PF	48.26 (±9.06)	51.80 (44.15–55.63)	25.01–57.54
RP	26.84 (±3.60)	27.96 (23.47–30.21)	21.23–30.21
BP	45.13 (±13.37)	46.68 (37.00–55.55)	21.68–62.00
GH	38.10 (±5.57)	38.92 (35.24–41.90)	26.08–46.07
MCS	41.04 (±7.42)	40.82 (35.28–44.58)	27.58–67.47
VT	50.82 (±8.58)	49.63 (43.69–56.31)	34.77–70.42
SF	47.57 (±11.29)	49.82 (42.30–57.34)	7.29–57.34
RE	22.74 (±6.77)	24.83 (17.87–24.83)	14.39–56.17
MH	48.38 (±8.31)	48.25 (45.64–53.48)	27.32–63.95

*BP* bodily pain, *GH* general health perceptions, *IQR* interquartile range, *MCS* mental component summary, *MH* mental health, *PCS* physical component summary, *PF* physical functioning, *RE* role limitations due to emotional problems, *RP* role limitations due to physical health problems, *SF* social functioning, *SD* standard deviation, *VT* vitality

The highest Skindex-29 scores were obtained within emotions (23.1 ± 8.5). Seven (17.5%) of patients had moderate or greater (≥32) and six (15%) patients mild (≥24) impairment of HRQoL in this domain. In 12 (30%) subjects functioning domain of Skindex-29 was affected, mildly (≥21) in 8, moderately (≥32) in 3, and severely (≥37) in 1 patient. But the vast majority of study participants had MCS and PCS scores of SF-36 below the average HRQoL, 92.5 and 82.5%, respectively. The lowest SF-36 scores were noted in RE (22.7 ± 6.8), RP 26.8 ± 3.6) and GH (38.1 ± 5.6).

**Fig. 1:**
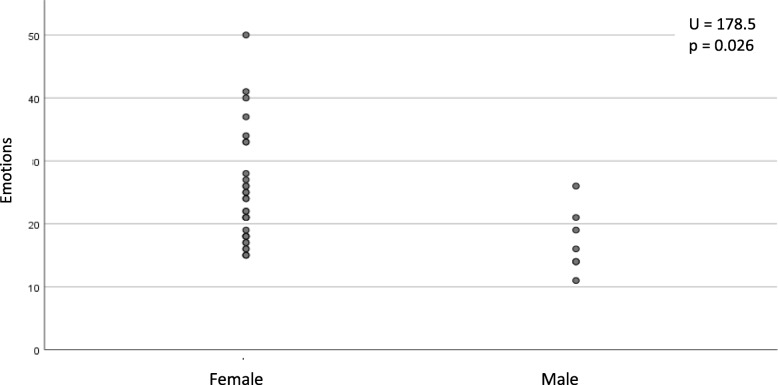
Relationship between scores on the emotion subscale of the Skindex-29 and gender of localized scleroderma patients

**Fig. 2:**
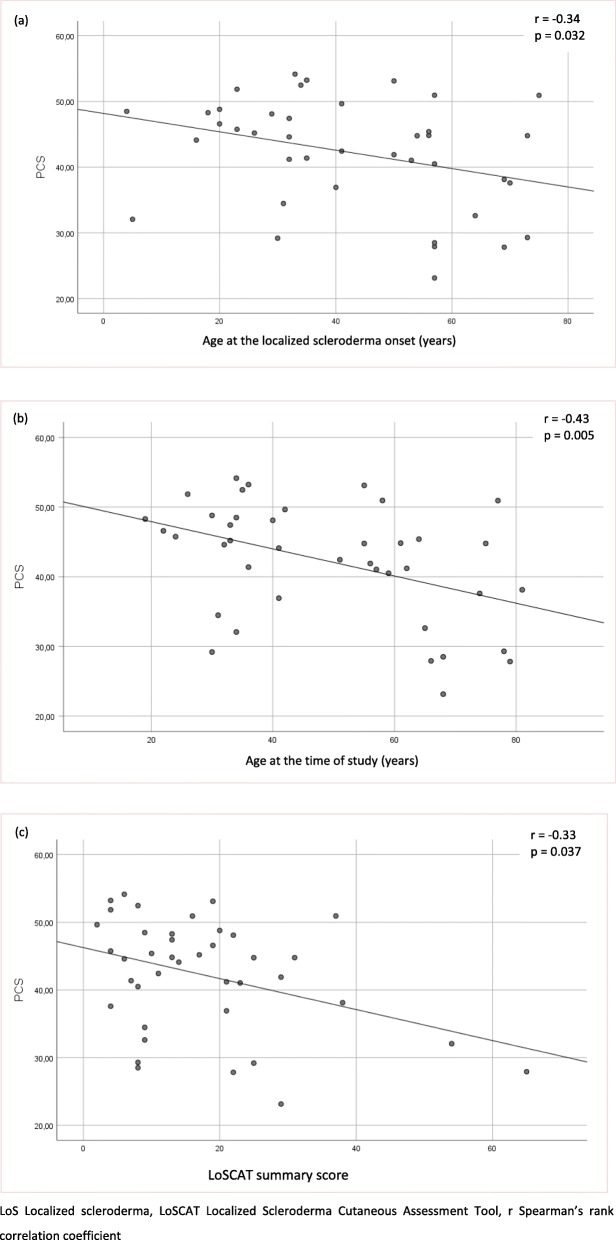
Associations of physical component summary (PCS) of the Short Form – 36 with age at the LoS onset (**a**), age at the time of study (**b**), and LoSCAT summary score (**c**). LoS Localized scleroderma, LoSCAT Localized Scleroderma Cutaneous Assessment Tool, r Spearman’s rank correlation coefficient

**Fig. 3:**
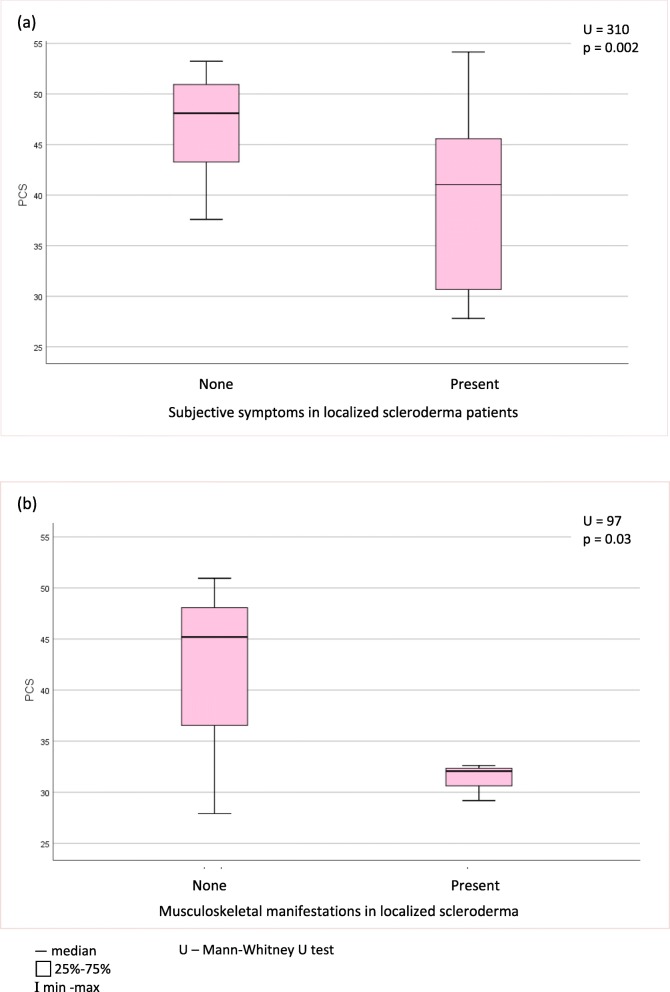
Associations of physical component summary (PCS) of the Short Form – 36 with the presence of subjective symptoms (**a**) and musculoskeletal manifestations (**b**) in localized scleroderma patients. U – Mann-Whitney U test

**Fig. 4:**
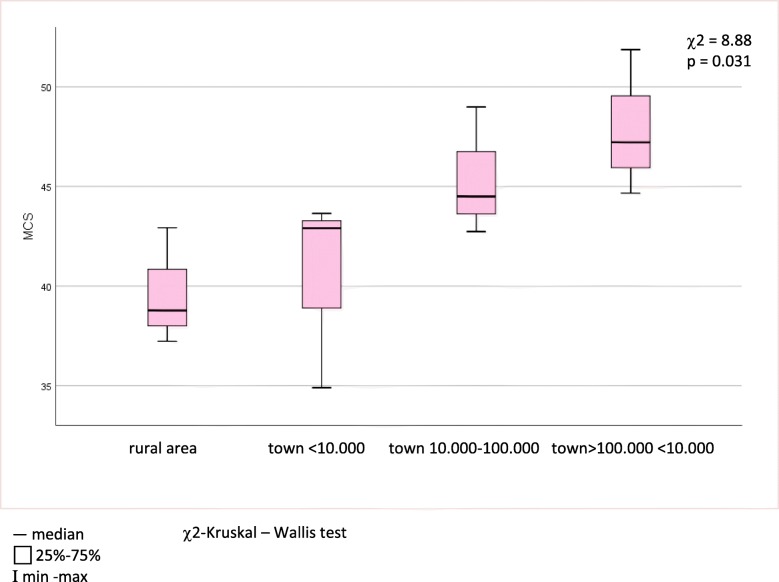
Relationship between mental component summary (MCS) of the Short Form – 36 and the place of residence of localized scleroderma patients

Relationships of demographic and clinical factors with HRQoL are presented in Tables 3, 4 and 5 and Figs. 1, 2, 3 and 4. Following univariate analysis, factors such as gender, age, place of residence, LoSCAT summary score, subjective symptoms, and extracutaneous manifestations - were identified as being significantly associated with HRQoL (*P* < 0.05). Female gender was a factor associated with worse Skindex-29 emotion subscale score (U = 178.5, *p* = 0.026). None of the other tested factors correlated with impaired HRQoL assessed by Skindex-29. The older age at the LoS onset (*r* = − 0.34, *p* = 0.032) and at the time of study (*r* = − 0.43, *p* = 0.005) were the demographic factors related with impairment of HRQoL in the PCS domain of SF-36. Place of residence affected the SF-36 MCS and patients who lived in urban areas had better HRQoL than those living in the rural areas (χ2 = 8,88, *p* = 0.031). Among clinical factors associated with worse SF-36 PCS, LoSCAT summary score (*r* = − 0.33, *p* = 0.037), subjective symptoms (U = 97, *p* = 0.03) and presence of extracutaneous manifestations (U = 310, *p* = 0.002) were found. Except for the age at the LoS onset and LoSCAT summary score, the other included variables remained significant (*p* < 0.05) as independent factors affecting HRQoL in the multiple linear regression analysis: the older age at survey, symptoms and musculoskeletal manifestations for the PCS, and the rural residence for the MCS.

**Table 3:** Associations of the Skindex 29 domains with sociodemographic and clinical characteristics of localized scleroderma patients

Possible factor	Statistical test	Symptoms		Functions		Emotions	
		result	p	result	p	result	P
Age at survey (years)	Spearman rank correlation	−0.12	0.48	−0.14	0.38	−0.09	0.60
Age at disease onset (years)		−0.17	0.29	−0.13	0.42	−0.69	0.67
Disease duration (years)		0.14	0.40	0.05	0.74	0.11	0.52
Body surface area affected by LoS		0.05	0.76	0.18	0.26	0.17	0.30
LoSCAT summary score		−0.01	0.95	0.22	0.18	0.10	0.54
Gender	U Mann-Whitney	101.5	0.63	141.5	0.36	**178.5**	**0.026**
Comorbid medical conditions		195.5	0.83	234.5	0.19	232.5	0.21
Musculoskeletal manifestations		28.0	0.16	29.5	0.19	43.5	0.55
Symptoms		146.5	0.15	158.0	0.26	148.5	0.17
Residence	Kruskal-Wallis	1.69	0.64	3.15	0.37	1.04	0.79
Education		2.47	0.48	1.95	0.58	0.48	0.92
Working		4.21	0.38	3.45	0.48	2.01	0.73
Marital status		4.32	0.36	7.37	0.12	7.84	0.10
Activity of the disease		4.51	0.21	0.85	0.84	1.17	0.76

The result of Spearman correlation test is r. The result of U Mann-Whitney test is U-statistics and of the Kruskal-Wallis is chi-square

**Table 4:** Associations of the Short Form – 36 with sociodemographic and clinical characteristics of localized scleroderma patients

Possible factor	Statistical test	PCS		MCS	
		Result*	P	result	P
Age at survey	Spearman rank correlation	**−0.43**	**0.005**	0.06	0.70
Age at disease onset (years)		**−0.34**	**0.032**	0.16	0.33
Disease uration (years)		−0.24	0.13	−0.13	0.44
Body surface area affected by LoS		−0.26	0.10	0.22	0.18
LoSCAT summary score		**−0.33**	**0.037**	0.19	0.25
Gender	U Mann-Whitney	102.0	0.65	75.0	0.15
Comorbid medical conditions		209.0	0.56	206.5	0.61
Musculoskeletal manifestations		**97.0**	**0.030**	74.0	0.36
Symptoms		**310.0**	**0.002**	210.0	0.79
Residence	Kruskal-Wallis	6.97	0.07	**8.88**	**0.031**
Education		2.97	0.40	0.88	0.83
Working		9.05	0.06	4.17	0.38
Marital status		4.88	0.30	2.61	0.63
Activity of the disease		1.05	0.79	5.92	0.12

*MCS* mental component summary, *PCS* physical component summary

The result of Spearman correlation test is r. The result of U Mann-Whitney test is U-statistics and of the Kruskal-Wallis is chi-square

**Table 5:** Multivariate regression analysis – dependent variables: Short Form – 36 physical component summary (a) and Short Form – 36 mental component summary (b)

**(a) Independent variable**	**Regression coefficient**	**Standard error**	**t-statistics**	**P**
Age at Survey	−0.40	0.12	−3.25	0.0025
Age at Disease Onset	0.19	0.12	1.65	0.1048
Symptoms (e.g. pruritus)	−8.13	1.99	−4.09	< 0.001
Musculoskeletal manifestations	−8.33	3.76	−2.21	0.0313
LoSCAT summary score	−0.010	0.08	−0.23	0.8203
**(b) Independent variable**	**Regression coefficient**	**Standard error**	**t-statistics**	**P**
Residence: urban, less than 10.000 of people	−0.07	3.54	−0.02	0.9837
Residence: urban, more than 10.000 and less than 100,000 of people	−0.16	2.23	−0.07	0.9416
Residence: urban, 100.000 or more of people	6.06	2.89	2.10	0.0387

## Discussion

In our study analyzing sociodemographic factors of LoS patients and their relationship with HRQoL, older age and female gender were the ones that most influenced this variable, followed by rural place of residence. Being older at survey and at disease onset negatively influenced the PCS of SF-36, which seems to be typical trait of physical functioning impairment with age [13]. According to Klimas et al. [5], patients may have difficulty discerning the impact of LoS in the context of multiple health concerns and coexistent diseases may have a significant effect across all the HRQoL measures (SF-36, Skindex-29 and DLQI). In contrast however, HRQoL of our patients was not impaired by the presence of comorbidities. It should be highligted that detailed information about LoS and its natural course was prepared and this information was made available to patients in the present study. Consistent with general population studies and earlier researches on LoS, female gender of our patients was associated with worse HRQoL [4, 9, 10]. At this point it should be added that LoS may result in a permanent skin and subcutaneous tissue damage causing aesthetic defect, affecting self-esteem and mental state particularly in women. Indeed, women participating in the presented study had higher scores of Skindex-29 emotion subscale than men as previously reported by Szramka-Pawlak et al. [9].

The rural place of residence as a predictor for the lower SF36 MCS among patients with LoS is firstly reported. On contrary, Zagozdzon et al. [12] found better mental health of Polish women aged 45–60 living in rural areas compared to those from urban settings. According to the authors [12], area of residence is strongly associated with environmental and psychosocial factors determining HRQoL in the spheres of physical and mental health. Poor subjective health, chronic diseases and low socioeconomic status may negatively affect the HRQoL of rural populations [12, 13]. Other sociodemographic factors evaluated firstly were education, marital and employment status, and none of them obtained significant results, neither in SF-36 nor in Skindex-29. Likewise, the effect of selected socioeconomic variables (race, regional income status and health insurance status) on HRQoL was investigated by Klimas et al. [5] and no significance for the functioning of LoS patients was shown.

Out of the clinical features of LoS assessed in our patients, only the LoSCAT summary score, subjective symptoms (itching, burning sensation, skin tightness or pain) and musculoskeletal involvement influenced the SF-36 PCS. Activity of the disease per se did not affect HRQoL of study subjects. Similarly to Klimas et al. results [5], the mLoSSI was more closely linked with Skindex-29 scores and SF-36 PCS than the LoSDI but no significant correlations were found, as previously described [17, 24]. Therefore, scores of the mLoSSI and LoSDI were added together and the relationship of the summary score with HRQoL was assessed. The sum of both indexes- the mLoSSI and LoSDI- seems to reflect general severity of skin lesions, as in the case of changes in the activity of LoS the signs of the damage usually occur. However, despite the correlation between the SF-36 PCS and the LoSCAT summary score, no significant impact of this score on Skindex-29 was revealed. The small sample size probably hindered the identifying of significant relationships from the data. Finding the itch, pain or other subjective symptoms as well as functional impairment among the factors influencing the HRQoL in adult LoS patients confirmed similar results from earlier reports [4–7, 11]. The SF-36 PCS was significantly affected by LoS accompanying complaints and extracutaneous features, while they did not significantly correlated with Skindex-29 score of the study subjects. Nonetheless, one third of them (i.e. 20% mildly, 10% moderately to severely) had impaired functioning domain.

There was no correlation between the LoS subtype and HRQoL in the present study. What is more, percentage of body surface area involved influenced neither skin disease-specific nor universal questionare scores. Some researchers, including the group from Poland signalized that Skindex-29 or DLQI score may not be linked with LoS subtype [4, 9]. However, it should be underlined that in the lager studies, patients with generalized LoS had significantly poorer HRQOL than those with the plaque or other subtype of the disease [5, 10]. LoS was also reported to have negative impact on HRQoL with the greater number of affected body segments or with greater lesion number [11]. Furthermore, neither disease duration nor number of recurrences correlated with HRQoL of our patients. Similar results for HRQoL and disease duration were described by Kroft et al. and Das et al. [4, 6]. Most importantly, further research should include larger groups of patients representing different characteristics.

Researches on HRQoL seem to be very important to the proper management of LoS. Some authors signalized the need for creating a specific LoS HRQoL instrument in order to meet the complexity of patient concerns within this disease [15, 25]. Our results demonstrated that the SF-36 is well-suited for application across health states in LoS. Skindex-29 - a skin disease specific instrument - did not capture all these aspects, whilst they are covered by the SF-36. The major limitation of this study, however, eluding to cross-sectional study design, small sample size, generalizability of the study population characteristics and predictor selection methods, was the lack of a control group. Nevertheless, this is the first study that showed relationship between the SF36 MCS of LoS patients and the place of residence. Therefore, we would like to highlight that the HRQoL in rural populations deserves further investigation because it may be an important factor in a development of a support strategies for patients. Unlike to earlier studies, the sum of both indexes- the mLoSSI and LoSDI, was correlated with the HRQoL scores. The LoSCAT summary score was linked to SF-36 PCS of our patients, apart from a subjective symptoms and musculoskeletal manifestations. This implies, that the global index may be an important instrument for assessment of skin involvement in LoS, if we are to come up with holistic approach to the patient.

## Conclusions

Older age, global skin involvement, presence of a subjective symptoms and extracutaneous manifestations may negatively affect the perceived physical health of adult patients with LoS. Female gender and rural residence may have negative impact on emotional or mental health, but further research is needed to clarify factors related to impaired HRQoL in these spheres.
